# Etiologic heterogeneity and a common imaging endpoint in reversible splenial lesion syndrome

**DOI:** 10.3389/fped.2026.1931158

**Published:** 2026-10-27

**Authors:** Yongmei Sun, Zongtao Mao, Shu Dong, Xuan Kan, Lihong Jiang, Qing Mu

**Affiliations:** 1 Department of Pediatrics, Tianjin Medical University General Hospital, Tianjin, China; 2 Department of Medical Cosmetology, Tianjin Medical University General Hospital Airport Hospital, Tianjin, China

**Keywords:** GFAP astrocytopathy, hyponatremia, RESLES, systemic homeostatic disturbance, viral infection

## Abstract

This brief research report describes eight children with RESLES treated at a single tertiary hospital. Etiologies included influenza A infection, respiratory syncytial virus with adenovirus coinfection, clinically suspected enterovirus-related herpangina, autoimmune glial fibrillary acidic protein astrocytopathy (GFAP-A), and one severe infantile encephalopathy with RESLES-like callosal involvement. We aimed to summarize the clinical, laboratory, EEG, and MRI features of these patients and to propose a practical framework in which diverse systemic or CNS insults converge on homeostatic disturbance and cytotoxic lesions of the corpus callosum (CLOCCs).

## 1 Introduction

Reversible splenial lesion syndrome (RESLES) is a clinical-radiological syndrome characterized by transient lesions involving the splenium of the corpus callosum (SCC), typically showing hyperintensity on diffusion-weighted imaging (DWI) with reduced apparent diffusion coefficient (ADC) values. RESLES overlaps with mild encephalitis/encephalopathy with a reversible splenial lesion (MERS), but RESLES is broader because similar lesions may occur with infection, metabolic or electrolyte disturbance, drug exposure or withdrawal, autoimmune disease and systemic illness (1–5). In children, this imaging pattern often appears during acute febrile or encephalopathic illness. Although the splenial lesion is usually reversible, correct interpretation is important because the underlying trigger may require targeted evaluation and treatment.

A central challenge is the coexistence of etiologic heterogeneity and radiological homogeneity. Viral encephalopathy, hyponatremia, metabolic acidosis, immune-mediated central nervous system (CNS) inflammation, dehydration, and critical illness are biologically different, yet they may converge on a similar SCC-restricted diffusion pattern (2, 3, 6, 7). Previous pediatric studies have emphasized infection-associated RESLES, favorable outcomes, and the value of follow-up MRI (8–11). However, small series remain useful when they compare individual triggers, laboratory abnormalities, treatment decisions, and early outcomes in detail.

This brief research report describes eight children with RESLES treated at a single tertiary hospital. Etiologies included influenza A infection, respiratory syncytial virus with adenovirus coinfection, clinically suspected enterovirus-related herpangina, autoimmune glial fibrillary acidic protein astrocytopathy (GFAP-A), and one severe infantile encephalopathy with RESLES-like callosal involvement. We aimed to summarize the clinical, laboratory, EEG, and MRI features of these patients and to propose a practical framework in which diverse systemic or CNS insults converge on homeostatic disturbance and cytotoxic lesions of the corpus callosum (CLOCCs).

## 2 Materials and methods

### 2.1 Study design and population

This retrospective single-center case series reviewed children admitted to the Department of Pediatrics, Tianjin Medical University General Hospital, from March 2022 to December 2025. Inclusion criteria were brain MRI evidence of a localized SCC lesion or SCC-predominant lesion, with or without additional brain or spinal involvement; radiological resolution on follow-up MRI; no previous history of epilepsy; and at least 6 months of clinical follow-up. Patients were excluded when the lesion persisted, imaging was more consistent with acute disseminated encephalomyelitis, multiple sclerosis spectrum disease, ischemia, tumor, trauma, or another primary callosal disorder, or when clinical or follow-up data were insufficient.

### 2.2 Clinical and laboratory data collection

Clinical information was extracted from inpatient and follow-up records. Variables included age, sex, prodromal symptoms, neurological manifestations, peak temperature, interval from symptom onset to initial MRI, pathogen testing, serum sodium and other electrolyte data, cytokines, cerebrospinal fluid (CSF) lactate dehydrogenase (LDH), CSF cell count and protein when available, autoimmune antibody testing, EEG, acute treatment, MRI follow-up interval, and neurological outcome. We specifically recorded markers of systemic or CNS homeostatic disturbance, including fever, dehydration, hyponatremia, low urine specific gravity, inflammatory cytokine elevation, CSF inflammation, and severe encephalopathy.

### 2.3 MRI assessment and RESLES classification

All patients underwent cranial MRI during the acute or subacute phase. Reviewed sequences included T1-weighted imaging, T2-weighted imaging, fluid-attenuated inversion recovery, DWI, and ADC maps. Type 1 RESLES was defined as an isolated SCC lesion. Type 2 RESLES was defined as SCC involvement accompanied by additional lesions in other parts of the corpus callosum, centrum semiovale, periventricular white matter, brainstem, spinal cord, or other regions (8–11). Follow-up MRI was used to confirm reversibility.

### 2.4 Statistical analysis

Because of the small sample size, only descriptive statistics were used. Categorical variables are reported as counts and percentages, and continuous variables as medians and ranges when appropriate. Individual cases were also described qualitatively to preserve clinically relevant differences among etiologies.

## 3 Results

### 3.1 General information and etiologic spectrum

Eight children met the inclusion criteria. Five were female and three were male. Age at onset ranged from 1.5 to 168 months, with a median of 82 months. The interval from symptom onset to initial cranial MRI ranged from 3 to 20 days, with a median of 4.5 days. Six patients had type 1 RESLES and two had type 2 RESLES. Seven children had fever or infection-related prodromal symptoms. Definite pathogen positivity was documented in five patients (62.5%): influenza A in four and respiratory syncytial virus combined with adenovirus in one. One additional patient had fever, sore throat, headache, and meningeal irritation with a clinical diagnosis of herpangina; although pathogen testing was negative, enterovirus-related infection could not be excluded. One patient had GFAP-A-related RESLES, and one afebrile infant developed callosal genu and splenial lesions during severe encephalopathy, respiratory failure, seizure activity, dehydration, and multisystem abnormalities (See Tables 1, 2).

**Table 1:** Summary of the main characteristics of pediatric RESLES cases in this series.

Item	Findings	Notes
Number of cases	8 cases (March 2022 to December 2025)	Single-center retrospective case series
RESLES classification	Type 1, 6 cases; type 2, 2 cases	Type 2 included GFAP-A-related multifocal involvement and infantile involvement of the corpus callosum genu and splenium
Infection-related cases	Definite pathogen positivity in 5 cases (62.5%); infection-related or highly suspected infection in 6 cases (75.0%)	Influenza A, 4 cases; RSV combined with adenovirus infection, 1 case; herpangina/suspected enterovirus infection, 1 case
Hyponatremia	4 cases	Nadir serum sodium, 110.2-127.5 mmol/L
GFAP-A-related disease	1 case	Suggestive of an autoimmune etiology
Elevated IL-6	6 cases	Concomitant IL-10 elevation in 3 cases
Imaging outcome	Lesions resolved on follow-up MRI	Follow-up MRI interval ranged from less than 3 days to approximately 3 months
Clinical outcome	Overall favorable	Follow-up ranged from 6 months to 3.5 years

**Table 2:** Clinical, laboratory, neuroimaging, and outcome data of pediatric RESLES cases in this series.

No.	Age/Sex	Pathogen	Prodromal symptoms	Initial symptoms	Peak body temperature	Interval from onset to cranial MRI	Serum sodium	Cytokines IL-6, IL-10	CSF LDH	Cranial MRI	Interval to second follow-up MRI	EEG	Initial diagnosis	Major treatments	Outcome
1	51 mo/F	Influenza A virus RNA positive	Fever with vomiting and abdominal pain	Somnolence, fatigue, and dizziness	Maximum temperature 39.8 °C, with 4–5 fever peaks per day	3 days	124.8 L	IL-6 27.31 H; IL-10 28.71 H	16 H	Abnormal signal in the splenium: patchy slightly long T1 and T2 signal with blurred margins; high signal on DWI.	Resolved on repeat MRI within less than 5 days	Normal	Callosal encephalopathy, influenza A, respiratory tract infection, acute gastritis, dehydration, and hyponatremia	Dexamethasone plus oseltamivir	No sequelae during 1 year of follow-up
2	167 mo/F	Influenza A virus antigen positive	Fever with dizziness, headache, and cough	Personality change, abnormal behavior, impaired memory and calculation ability, staring episodes, irrelevant answers, and one episode of loss of consciousness	Maximum temperature 39.9 °C, with 1 fever peak per day	7 days	Normal	IL-6 1.14; IL-10 NP	15.6 H	A round lesion in the splenium with slightly long T1 and T2 signal and blurred margins.	Resolved on repeat MRI after approximately 2.5 months	Paroxysmal medium- to high-amplitude theta activity in the frontal regions during sleep	Influenza-associated encephalopathy, bronchitis, and rhabdomyolysis	Methylprednisolone, intravenous immunoglobulin, and oseltamivir	No sequelae during 2 years of follow-up
3	94 mo/F	Adenovirus DNA positive; respiratory syncytial virus antigen positive	Fever with headache and vomiting	Depressed mood and tearfulness	Maximum temperature 39.3 °C, with 2–3 fever peaks per day	3 days	127.5 L	IL-6 42.90 H; IL-10 15.65 H	12 H	Patchy high signal in the splenium on DWI.	Resolved on repeat MRI after 1 month	Normal	Infection-associated encephalopathy, adenovirus infection, respiratory syncytial virus infection, and hyponatremia	Methylprednisolone and ganciclovir	No sequelae during 2 years of follow-up
4	70 mo/M	Negative	Fever with headache and sore throat	Neck stiffness	Maximum temperature 39.4 °C; after antipyretic treatment, temperature remained 38–38.5 °C and was difficult to normalize	5 days	Normal	IL-6 27.8 H; IL-10 NP	13 H	A nearly round abnormal signal in the midline splenium, showing slightly long T1 and T2 signal, slightly high signal on T2-FLAIR, and marked high signal on DWI with mildly blurred margins.	Resolved on repeat MRI after slightly more than 1 month	Normal	Infection-associated encephalopathy and herpangina	Dexamethasone	No sequelae during nearly 3 years of follow-up
5	144 mo/F	Influenza A virus antigen positive	Fever	Incoherent speech	Maximum temperature 39.5 °C, with 2 fever peaks per day	3 days	Normal	IL-6 22 H; IL-10 NP	NP	A round slightly long T1 signal lesion in the splenium, showing high signal on DWI.	Resolved on repeat MRI after approximately 1 month	Normal	Influenza A-associated encephalopathy	Methylprednisolone and oseltamivir	No sequelae during 6 months of follow-up
6	32 mo/M	Influenza A virus antigen positive	Fever and cough	Vomiting, one afebrile seizure, and subsequent depressed mood	Low-grade fever after seizure (37.6–38.2 °C)	4 days	117 L	IL-6 13.85 H; IL-10 NP	NP	Small patchy slightly long T2 signal lesion in the splenium, showing high signal on DWI.	Resolved on repeat MRI after approximately 3 months	Normal	Influenza A-associated encephalopathy and hyponatremia	Methylprednisolone and two courses of oseltamivir	No sequelae during 3 years of follow-up
7	168 mo/F	Negative	Fever with headache and dizziness	Moderate anxiety and mild depression, personality change, diplopia, difficulty with urination and defecation, urinary retention, and neck stiffness	Maximum temperature 40.4 °C, with 2–5 fever peaks per day	12 days	110.2 L	IL-6 7.96 H; IL-10 8.40 H	55.0 H	Multiple punctate and patchy slightly high-signal lesions on DWI in the bilateral centrum semiovale, bilateral periventricular regions adjacent to the anterior and posterior horns of the lateral ventricles, and corpus callosum, with blurred margins. Signal in the medulla oblongata and cervical spinal cord was heterogeneous, with blurred patchy slightly long T2 signal.	Resolved on repeat MRI within less than 3 full days	Scattered medium-amplitude slow waves during wakefulness; borderline AEEG	GFAP-A and hyponatremia	Pulse methylprednisolone for 3 days followed by gradual tapering; intravenous immunoglobulin	No sequelae during 8 months of follow-up
8	1.5 mo/M	Negative	Intermittent staring, drooling, and poor feeding	Convulsions, weak crying, refusal to feed, somnolence, decreased primitive reflexes, and subsequent coma	No fever	20 days	Normal	IL-6 5.1; IL-10 NP	29 H	Small patchy slightly high-signal lesions in the genu and right splenium of the corpus callosum on DWI.	Resolved on repeat MRI after approximately 1.5 months	Ambulatory EEG showed paroxysmal high-amplitude broad sharp waves involving all leads, right-side predominant, lasting up to approximately 7 s; scattered focal sharp waves were also seen, most prominent in the central-parietal-occipital regions and left-side predominant	Severe encephalopathy, cerebral herniation, type II respiratory failure, epileptic seizures, abnormal liver function, anemia, hypoproteinemia, ascites, pericardial effusion, and mild dehydration	Dexamethasone, prednisone, intravenous immunoglobulin, mechanical ventilation, and antiseizure medications	No sequelae during 3.5 years of follow-up

Cases are grouped by clinical/etiologic pattern; original case numbers are retained for consistency with the manuscript. mo, months; F, female; M, male; NP, not performed. H/L indicate values above or below the corresponding reference range; values without H or L are within the reference range; “Normal” indicates a value within the reference range.

Units and reference values: serum sodium, mmol/L (136–145); cytokines, pg/mL (IL-6 < 7; IL-10 < 4.91); CSF LDH, U/L (<10.5).

Additional blood/urine/coagulation findings not shown in the main columns: Case 2, urine specific gravity 1.002 (1.005–1.030); Case 7, plasma osmolality 252 mOsm/kg H₂O (275–305), urine sodium 59.7 mmol/L, urine osmolality 670 mOsm/kg H₂O, serum uric acid 59 μmol/L (140–414) and D-dimer 4,166 ng/mL (0–500).

Additional CSF and immune-related findings not shown in the main columns: Case 2, oligoclonal bands negative in CSF and serum; Case 7, CSF pressure 478 mmH₂O, Pandy test weakly positive (±), leukocytes 234 × 10^6^/L (polymorphonuclear cells 12%, mononuclear cells 88%), CSF protein 0.76 g/L (0.20–0.40), CSF glucose 2.8 mmol/L(blood glucose7.2), NGS negative, CSF GFAP antibody positive 1:32, and serum GFAP antibody positive 1:10; Case 8, CSF Pandy test 1+, leukocytes 8 × 10^6^/L, and CSF protein 0.81 g/L; Cases 5 and 6, CSF examination not performed.

### 3.2 Laboratory and immune-inflammatory findings

Hyponatremia occurred in four patients, with nadir serum sodium values of 124.8 mmol/L, 127.5 mmol/L, 117 mmol/L and 110.2 mmol/L. The most severe hyponatremia occurred in the GFAP-A case, which also showed syndrome of inappropriate antidiuretic hormone secretion (SIADH) (low plasma osmolality, low serum uric acid, elevated urine sodium and urine osmolality). Among infection-associated patients, hyponatremia was observed in two influenza A cases and one adenovirus-respiratory syncytial virus coinfection case, suggesting that electrolyte disturbance may overlap with infection or autoimmunity rather than represent a separate etiology.

Cytokine abnormalities were frequent. Interleukin-6 (IL-6), a proinflammatory cytokine, was elevated in six patients, and three of these also had elevated interleukin-10 (IL-10), an anti-inflammatory and immunoregulatory cytokine. CSF lactate dehydrogenase (LDH) was measured in six patients and exceeded the reference upper limit in all tested cases. Elevated CSF LDH can indicate tissue injury, cellular breakdown, or inflammation within the central nervous system. The GFAP-A case had the most prominent inflammatory pattern, including markedly elevated CSF pressure, mononuclear cell-predominant pleocytosis, increased CSF protein, and positive GFAP antibodies in both serum and CSF. These findings indicate overlapping inflammatory, osmotic, and cellular stress signals in pediatric RESLES.

### 3.3 Clinical manifestations

Clinical manifestations were heterogeneous. Seven children had fever or an infection-related prodrome, with peak temperatures generally at or above 39.3 °C and reaching 40.4 °C in one patient. Prodromal symptoms included fever with vomiting, abdominal pain, headache, cough, or sore throat. Neurological manifestations included fatigue, dizziness, somnolence, incoherent speech, altered consciousness, personality change, behavioral abnormality, depressed mood, tearfulness, and impairment of memory or calculation. Two patients had convulsions or afebrile seizures, and two had neck stiffness. The GFAP-A case also developed diplopia, difficulty with urination and defecation, urinary retention, and brainstem or spinal cord involvement, consistent with meningoencephalomyelitis.

Most infection-associated type 1 cases improved rapidly after supportive and etiologic treatment and had no persistent focal neurological deficits. In contrast, the two type 2 cases were clinically more complex. The GFAP-A patient had autoimmune CNS inflammation with multifocal involvement and severe hyponatremia. The infant had severe encephalopathy with respiratory failure, epileptic seizures, cerebral herniation, hepatic dysfunction, anemia, hypoproteinemia, ascites, pericardial effusion, and mild dehydration. These observations suggest that extrasplenial lesions may mark greater systemic severity or stronger CNS inflammatory and metabolic stress, although larger cohorts are needed.

### 3.4 Neuroimaging and EEG features

All children had splenial or splenial-predominant MRI abnormalities. Typical findings included T2-weighted or FLAIR hyperintensity, DWI hyperintensity, and reduced ADC values, compatible with restricted diffusion related to cytotoxic injury or cytotoxic edema (3). Six patients had isolated SCC lesions. In the GFAP-A case, MRI showed multifocal DWI hyperintensities involving the bilateral centrum semiovale, periventricular regions adjacent to the anterior and posterior horns of the lateral ventricles, and corpus callosum, with additional abnormal signal in the medulla oblongata and cervical spinal cord. In the infant with severe encephalopathy, small patchy DWI hyperintensities involved the genu and right splenium of the corpus callosum. Follow-up MRI confirmed lesion resolution in all cases within less than 3 days to approximately 3 months (See Figure 1).

**Figure 1:**
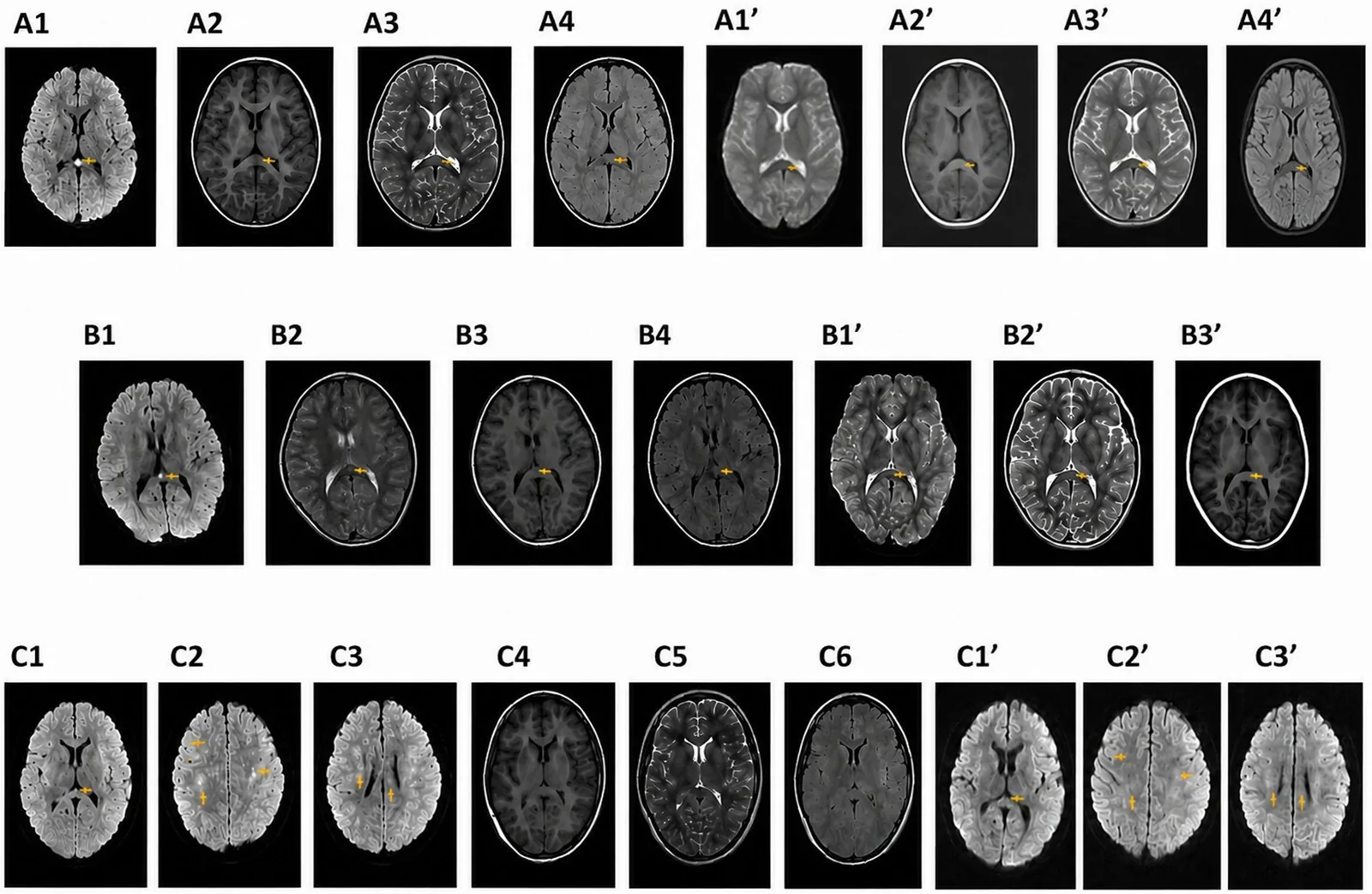
Representative cranial MRI findings before and after treatment in pediatric RESLES. A1-A4 and A1′-A4′ show the first MRI set of Case 1 before and after treatment, respectively. The pretreatment splenial lesion (arrows) showed DWI hyperintensity, slight T1-FLAIR hypointensity, and slight T2WI/T2-FLAIR hyperintensity, with complete resolution on follow-up MRI. B1-B4 and B1′-B3′ show Case 4 before and after treatment, respectively. The pretreatment ovoid midline splenial lesion (arrows) showed marked DWI hyperintensity with mild T1-FLAIR hypointensity and T2WI/T2-FLAIR hyperintensity, and no residual abnormal signal was seen on follow-up MRI. C1-C6 and C1′-C3′ show Case 7 before and after treatment, respectively. Pretreatment DWI showed multiple punctate and patchy hyperintense lesions involving the corpus callosum, bilateral centrum semiovale, and bilateral periventricular white matter (arrows), without corresponding abnormalities on T1-FLAIR, T2WI, or T2-FLAIR; follow-up DWI showed complete resolution.

EEG was normal in five patients. Three patients had abnormalities, including paroxysmal medium- to high-amplitude theta activity in the frontal regions during sleep, scattered medium-amplitude slow waves during wakefulness with borderline amplitude-integrated EEG, and paroxysmal high-amplitude broad sharp waves involving multiple leads. EEG abnormalities were more evident in patients with impaired consciousness, seizure-like episodes, or severe encephalopathy.

### 3.5 Treatment and clinical outcomes

Treatment was individualized according to suspected etiology and severity. All four influenza A-associated patients received oseltamivir. The patient with adenovirus and respiratory syncytial virus coinfection received empiric off-label ganciclovir in view of the severity of illness, alongside corticosteroids and supportive care. Clinical improvement was observed during the combined treatment; however, the specific contribution of ganciclovir could not be determined. All patients received corticosteroids during the acute phase, and three with more severe disease or stronger immune-inflammatory features received intravenous immunoglobulin. The patient with GFAP-A positivity received pulse methylprednisolone followed by gradual tapering and also received intravenous immunoglobulin. The infant with severe encephalopathy required mechanical ventilation and antiseizure therapy in addition to corticosteroids and intravenous immunoglobulin. Supportive care included correction of electrolyte disturbance and maintenance of fluid and internal milieu stability.

During 6 months to 3.5 years of follow-up, no definite neurological sequelae were documented. This favorable outcome is consistent with the usual reversibility of RESLES, particularly type 1 lesions. Nevertheless, seizures, abnormal EEG, severe hyponatremia, autoimmune inflammation, and type 2 involvement indicate that radiological reversibility should not be equated with a benign acute illness. Neurological, cognitive, behavioral, and developmental surveillance remains appropriate, especially for infants and children with severe presentations.

## 4 Discussion

### 4.1 Etiologic significance and shared pathophysiology

This series supports the view that pediatric RESLES is a shared imaging phenotype rather than a single disease entity. Infection was the most frequent association, with influenza A accounting for four of five pathogen-positive cases, but the cohort also included suspected enterovirus-related illness, autoimmune GFAP-A, severe hyponatremia, dehydration, and critical systemic illness. These observations emphasize that the splenial lesion should be interpreted as a radiological stress signal. The central splenium contains densely packed glial cells and callosal axons. Proposed explanations for its transient diffusion restriction include cytotoxic swelling of astrocytes and neurons and intramyelinic fluid shifts involving myelin and oligodendrocytes in response to inflammatory and osmotic disturbances. These mechanisms remain incompletely established; restricted diffusion alone does not demonstrate demyelination or permanent injury to commissural fibers (8, 12).

The laboratory profile strengthens this interpretation. IL-6 elevation, occasional IL-10 elevation, CSF LDH elevation, hyponatremia and syndrome of inappropriate antidiuretic hormone suggest that inflammatory, osmotic, and cellular stress pathways may operate together. Influenza-associated MERS has previously been linked to early cytokine elevation, including IL-10 and interferon-gamma (13). Low sodium has also been reported in pediatric MERS/RESLES and may contribute to cellular swelling in vulnerable white matter tracts (4). Clinically, serum sodium should be monitored dynamically, particularly when fever, vomiting, poor intake, dehydration, altered urine output, or severe systemic illness is present.

The GFAP-A case shows that autoimmune inflammation can also produce a RESLES phenotype. GFAP-A is an autoimmune astrocytopathy presenting with meningitis, encephalitis, myelitis, or meningoencephalomyelitis, and CSF GFAP-IgG is diagnostically important (14–16). Previous reports have described GFAP-A overlapping with MERS or RESLES (17–20). In our patient, elevated intracranial pressure, CSF pleocytosis, increased protein, GFAP antibody positivity in serum and CSF, diplopia, urinary retention, and brainstem-spinal cord involvement all supported a broader autoimmune CNS process. Autoimmune antibody testing should therefore be considered when RESLES is accompanied by meningeal signs, spinal cord or brainstem involvement, atypical course, negative routine pathogen tests, or disproportionate CSF inflammation.

Together, these findings support a homeostatic disturbance-SCC focal cytotoxic edema model. Infection, autoimmunity, hyponatremia, dehydration, metabolic acidosis, hypoxia, and severe systemic illness are not mutually exclusive explanations; they may interact through cytokine release, neurovascular unit dysfunction, blood-brain barrier alteration, astrocyte injury, osmotic imbalance, energy stress, and abnormal water transport (7, 21, 22). This model is clinically useful because it explains why different triggers may share a common imaging endpoint while still requiring different etiologic investigations and treatments.

### 4.2 Diagnostic and therapeutic implications

A practical diagnostic approach should begin by confirming a compatible MRI pattern: a discrete splenial or splenial-predominant lesion with restricted diffusion, absence of a destructive structural process, and resolution on follow-up imaging. The next step is to search for an infectious syndrome. Respiratory viral testing is especially relevant during influenza season, while adenovirus, respiratory syncytial virus, and enterovirus should be considered according to symptoms. Correctable internal milieu disturbances should be assessed early, including serum sodium, serum osmolality when available, urine output, urine specific gravity, urine osmolality, and urine sodium. Lumbar puncture is appropriate when meningitis, encephalitis, autoimmune inflammation, or intracranial hypertension is suspected. CSF pressure, cell count, protein, glucose, LDH, pathogen testing, oligoclonal bands, and autoimmune antibodies can help direct management. EEG should be integrated when seizures, altered consciousness, or fluctuating mental status occur.

There is no single disease-specific treatment for RESLES. Management should target the trigger and the child's severity rather than the splenial lesion alone. In infection-associated cases, pathogen-directed therapy and supportive care are central. Electrolyte and fluid disturbances should be corrected carefully, because persistent hyponatremia poses neurological risks, whereas overly rapid correction, particularly in chronic hyponatremia, can cause osmotic demyelination syndrome (ODS), including central pontine myelinolysis. Corticosteroids and intravenous immunoglobulin were used in this series, especially in more severe or immune-inflammatory cases. However, because this retrospective series had no control group and most patients received steroids, it cannot determine whether immunotherapy accelerated MRI resolution or improved outcome. Routine immunotherapy for mild infection-associated type 1 RESLES remains uncertain and should be individualized. Treatment priorities should include infection control, seizure management, intracranial pressure control, respiratory support when needed, correction of electrolyte and metabolic abnormalities, and immunotherapy when autoimmune inflammation is likely.

### 4.3 Prognosis, follow-up, and limitations

All children had radiological resolution and no definite neurological sequelae during available follow-up, supporting the generally favorable prognosis of pediatric RESLES, especially isolated SCC lesions. Prognosis should nevertheless be stratified by clinical severity. Children with seizures, abnormal EEG, profound hyponatremia, marked CSF abnormalities, autoimmune disease, type 2 lesions, or critical illness require closer follow-up than children with mild influenza-associated type 1 RESLES. Follow-up should include neurological examination, review of seizure recurrence, screening for cognitive or school difficulties, and assessment of behavioral or emotional change. Infants may require developmental surveillance because subtle deficits may emerge later. Repeat MRI is useful to document reversibility, but clinical recovery remains the primary outcome.

This study has several limitations. The sample was small, and the retrospective single-center design limits generalizability. Testing was not standardized across patients, especially for cytokines, CSF LDH, autoimmune antibodies, EEG follow-up, and timing of repeat MRI. Treatment was heterogeneous, preventing causal inference about hyponatremia, cytokine elevation, CSF LDH, or specific therapies. The apparent association between type 2 lesions and greater severity is hypothesis-generating only. Multicenter prospective studies with standardized laboratory timing, MRI protocols, severity scoring, neuropsychological assessment, and long-term developmental follow-up are needed.

## 5 Conclusion

Pediatric RESLES is a heterogeneous clinical-radiological syndrome characterized by reversible restricted diffusion involving the SCC and related white matter pathways. In this series, viral infection, particularly influenza A, was the most frequent association, while hyponatremia, cytokine elevation, CSF LDH abnormality, autoimmune GFAP-A, and systemic homeostatic disturbance appeared to act as overlapping contributors. The proposed homeostatic disturbance model provides a practical framework for understanding how diverse triggers may converge on focal cytotoxic edema in the SCC. Pediatric management should combine MRI recognition with systematic etiologic screening, electrolyte and fluid monitoring, autoimmune testing in atypical cases, and follow-up imaging and neurological surveillance.

## Data Availability

The original contributions presented in the study are included in the article/Supplementary Material, further inquiries can be directed to the corresponding author/s.
